# Multiomics integration of single-cell transcriptomics and bulk sequencing data identifies key biomarkers and predictive models for IBD subtype classification

**DOI:** 10.3389/fmed.2026.1729642

**Published:** 2026-03-19

**Authors:** Linghui Song, Zian Chen, Dongmei Yao, Huihui Ma, Quanxu Wang, Jiale Yao, Kaini Li, Liwei Liu, Qian Gu, Ruoying Ding, Luwei Jia, Zhixu Lu, Chuanjie Yang

**Affiliations:** 1 Department of Gastroenterology, The Second Hospital of Hebei Medical University, Hebei Key Laboratory of Gastroenterology, Hebei Institute of Gastroenterology, Hebei Clinical Research Center for Digestive Diseases, Shijiazhuang, Hebei, China; 2 Hebei Medical University, Shijiazhuang, Hebei, China

**Keywords:** biomarkers, Crohn’s disease, inflammatory bowel disease, machine learning, single-cell RNA sequencing, ulcerative colitis

## Abstract

**Introduction:**

Inflammatory bowel disease (IBD), comprising ulcerative colitis (UC) and Crohn’s disease (CD), represents a diagnostic challenge owing to its overlapping clinical features. Precise molecular characterization is essential for improved subtype classification and the development of reliable diagnostic tools.

**Methods:**

We integrated peripheral blood single-cell RNA sequencing (scRNA-seq) data (GSE125527) and bulk sequencing data (GSE75214) from UC and CD patients to identify molecular patterns distinguishing the two subtypes through cross-dataset integration analysis. Machine learning algorithms and network analysis were applied to identify hub genes and construct diagnostic models. Biomarker robustness was evaluated using an independent validation dataset (GSE179285), followed by immunohistochemical (IHC) confirmation in clinical samples.

**Results:**

Single-cell analysis revealed distinct immune signatures: UC patients exhibited IL-17A + effector memory T cells and enriched leukocyte migration pathways, whereas CD patients were characterized by IL-1β-producing immune cells and B cell activation processes. Cross-dataset integration identified 43 consistently dysregulated genes and 10 hub genes (THBS1, PLAUR, KLF4, CD36, CD44, CXCR4, FOS, S100A9, ANXA1, and TIMP1), spanning immune response, transcriptional regulation, metabolism, and tissue remodeling functions. An 18-gene machine learning model achieved an area under the curve (AUC) of 0.73 in independent validation. Notably, LDHB, MGAT4A, and PSME2 emerged as uniquely powerful biomarkers with limited prior reporting in UC/CD classification. IHC validation confirmed subtype-specific expression, with GZMA and PSME2 preferentially elevated in CD, and LDHB and FOSB preferentially elevated in UC.

**Discussion:**

This multiomics approach revealed robust molecular signatures distinguishing UC from CD, demonstrating moderate-to-good diagnostic capability (AUC = 0.73). The findings illuminate fundamental differences in immune regulation, metabolism, and tissue remodeling between IBD subtypes, providing a promising foundation for blood-based diagnostic tool development. Prospective clinical validation is warranted before implementation in routine practice.

## Introduction

Inflammatory bowel disease (IBD), which primarily comprises ulcerative colitis (UC) and Crohn's disease (CD), represents a group of chronic inflammatory conditions affecting millions of individuals worldwide, with incidence rates continuing to increase globally (1, 2). The global burden of IBD has increased substantially, with prevalence rates reaching 0.3% in North America and Europe (3). These conditions share overlapping clinical presentations, including abdominal pain, diarrhea, and systemic inflammation, but they exhibit distinct pathophysiological mechanisms and treatment responses that necessitate accurate differential diagnosis (4, 5). Current diagnostic approaches rely heavily on invasive endoscopic procedures, radiological imaging, and histopathological examination, which are associated with patient discomfort, potential complications, and significant health care costs (6, 7). The heterogeneous nature of IBD, combined with the lack of definitive biomarkers, often leads to diagnostic delays and misclassification, particularly when distinguishing between UC and CD in colonic disease (8, 9).

The advent of single-cell RNA sequencing (scRNA-seq) and multiomics integration has revolutionized the understanding of complex diseases by enabling unprecedented resolution of cellular heterogeneity and molecular mechanisms (10, 11). Recent studies have demonstrated the power of single-cell transcriptomics in IBD research, revealing distinct immune cell populations and pathway dysregulation patterns between UC and CD (12–14). These investigations have revealed cell type-specific signatures, including those of IL-17A + T cells in UC and IL-1β + immune cells in CD, providing insights into disease-specific inflammatory cascades (15, 16). The gut microbiome also plays a crucial role in IBD pathogenesis, with distinct microbial signatures associated with different disease subtypes (17). However, most existing studies have focused primarily on intestinal tissue samples, which require invasive procedures and may not reflect systemic immune alterations detectable in more accessible sample types. The integration of single-cell data with bulk sequencing approaches offers opportunities to validate findings across platforms and identify robust biomarkers that are reproducible across diverse patient populations.

Peripheral blood represents an ideal biospecimen for IBD biomarker development because of its accessibility and potential for reflecting systemic immune dysregulation associated with intestinal inflammation (18). Blood-based screening offers significant advantages over current diagnostic methods, including noninvasive sample collection, reduced patient burden, and the potential for repeated monitoring throughout disease progression and treatment response (19, 20). Previous studies have demonstrated that peripheral blood immune cells undergo significant transcriptomic changes in IBD patients, suggesting that blood-based molecular signatures may serve as surrogate markers for intestinal inflammation (21–23). The integration of machine learning approaches with multiomics data provides opportunities to develop robust predictive models that can distinguish between UC and CD with moderate-to-good discriminatory performance, potentially transforming IBD diagnosis from a predominantly invasive process to a rapid, blood-based screening approach.

In the present study, we developed a comprehensive multiomics integration framework by combining peripheral blood single-cell transcriptomics with bulk sequencing data to identify robust molecular biomarkers and develop predictive models for IBD subtype classification. Importantly, while the diagnostic model was derived from peripheral blood transcriptomic signatures—reflecting systemic immune dysregulation—we validated that the identified genes represent shared disease-associated molecular programs detectable in both circulating immune cells and intestinal tissue. This cross-compartment validation approach ensures that the blood-based biomarkers reflect fundamental disease mechanisms rather than compartment-specific expression artifacts. We systematically analyzed single-cell RNA sequencing data from the GSE125527 dataset, as well as bulk RNA sequencing data from the GSE75214 and GSE179285 datasets, to characterize distinct cellular and molecular landscapes distinguishing UC from CD. Through rigorous cross-dataset validation, we identified 43 consistently dysregulated genes, and we developed an 18-gene diagnostic signature using machine learning algorithms that achieved an AUC of 0.73 in independent validation, demonstrating moderate-to-good discriminatory performance in retrospective analysis. The identified biomarkers span four key functional domains, namely, immune response, transcriptional regulation, cellular metabolism, and tissue remodeling. We validated the transcriptomic findings through immunohistochemical analysis of patient tissue samples, and we performed a comprehensive immune infiltration analysis to elucidate the underlying mechanisms. The present multidimensional approach provides novel insights into molecular mechanisms distinguishing UC from CD while establishing a robust diagnostic framework with significant potential for clinical translation in IBD management.

## Materials and methods

### Data collection and preprocessing

**Figure 1:**
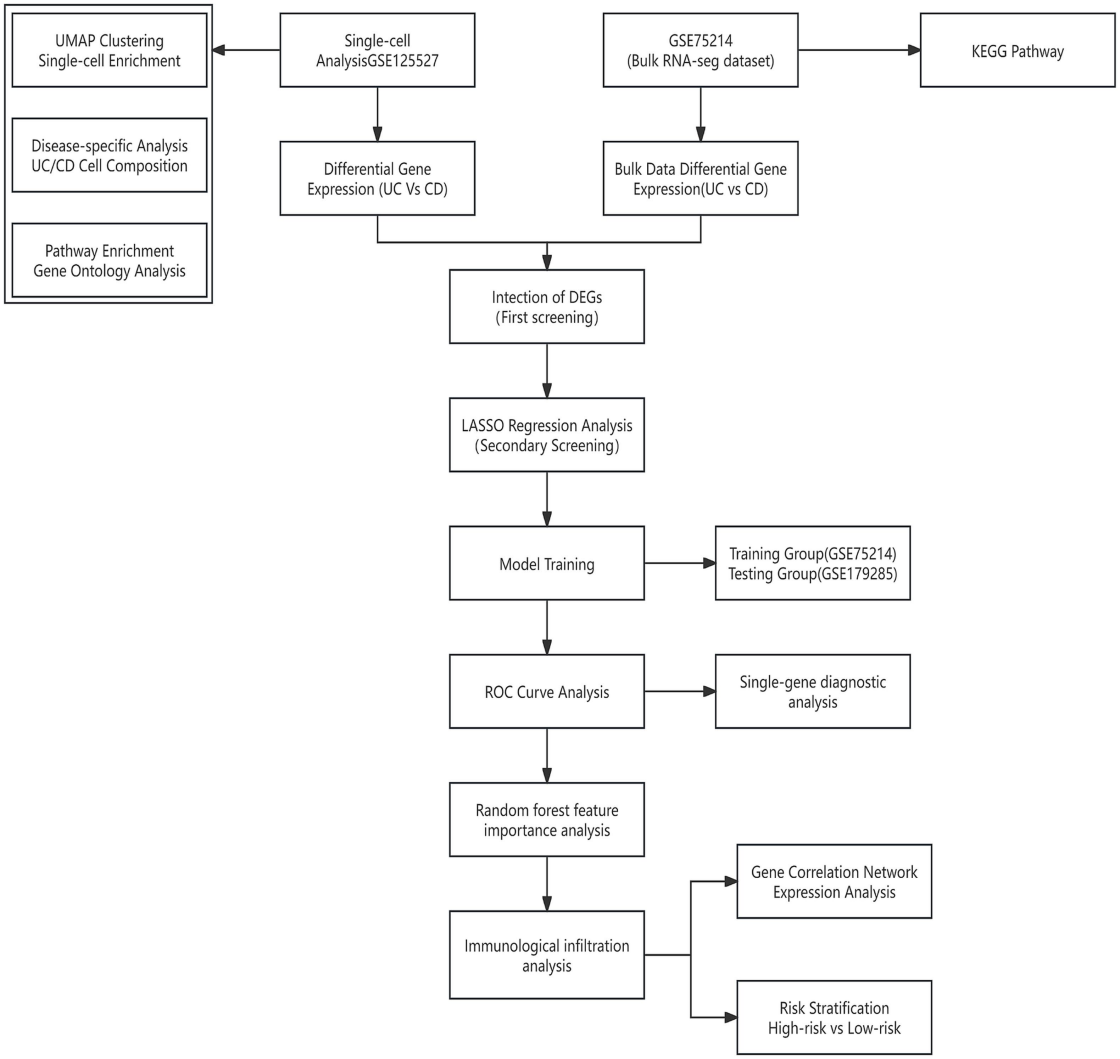
Comprehensive bioinformatics workflow for IBD subtype classification using multidataset integration and machine learning approaches. The workflow demonstrates the integration of single-cell RNA sequencing data (GSE125527) from the peripheral blood of both UC and CD patients with bulk transcriptomic datasets (GSE75214 for training; GSE179285 for validation) through differential expression analysis, cross-dataset validation, LASSO regression modeling, and immunohistochemical validation to identify an 18-gene diagnostic signature distinguishing ulcerative colitis from Crohn’s disease.

This retrospective bioinformatics study utilized publicly available gene expression datasets from inflammatory bowel disease (IBD) patients, including both patients with ulcerative colitis (UC) and those with Crohn's disease (CD) (Figure 1). Patient samples were derived from three primary datasets—GSE75214, GSE125527, and GSE179285—obtained from the Gene Expression Omnibus (GEO) database (Table 1) (24).

**Table 1:** Characteristics of GEO datasets used in this study.

GSE ID	Participants (CD/UC)	Analysis type	Platform	Year	Sex (male: female)	Included in the RRA analysis	Network address
GSE75214	59/74	Array	GPL6244	2017	Not determined	Yes	https://www.ncbi.nlm.nih.gov/geo/query/acc.cgi?acc=GSE75214
GSE179285	37/23	Array	GPL6480	2021	Not determined	No	https://www.ncbi.nlm.nih.gov/geo/query/acc.cgi?acc=GSE179285
GSE125527	7/7	Single-cell Sequencing	GPL20301	2020	Not determined	No	https://www.ncbi.nlm.nih.gov/geo/query/acc.cgi?acc=GSE125527

### Patient cohort characteristics and selection criteria

To ensure anatomical comparability between UC and CD for accurate subtype classification, the present analysis focused exclusively on colonic involvement. All UC patients included had colonic disease (UC is inherently limited to the colon), whereas for CD patients, only those with documented colonic involvement were included in the comparative analyses. Disease activity status was confirmed as active inflammatory disease in all included samples on the basis of endoscopic and histological criteria documented in the original studies. Samples from patients with quiescent or inactive disease were excluded to maintain consistency in the inflammatory state across comparisons.

### Tissue-specific analysis strategy

Given that UC affects only the colon and CD can involve any segment of the gastrointestinal tract, we implemented strict tissue-specific selection criteria. For the GSE75214 and GSE179285 bulk datasets, only colonic tissue samples were included in the UC vs. CD comparative analysis. Ileal CD samples were analyzed separately but were excluded from the direct UC vs. CD comparisons to prevent confounding due to anatomical differences. This approach ensured that the identified biomarkers reflected disease subtype differences rather than tissue-specific expression patterns.

### Sample selection and anatomical considerations

To address the inherent anatomical differences between UC (limited to the colon) and CD (which can affect any GI tract segment), we implemented rigorous tissue-specific selection criteria.

For the colonic UC vs. colonic CD comparison, the primary comparative analyses included only colonic tissue samples from both disease groups (UC: all samples inherently colonic; CD: only samples with documented colonic involvement). For the ileal CD analysis, ileal CD samples were analyzed separately to characterize CD-specific signatures but were excluded from direct UC vs. CD comparisons to prevent anatomical confounding. For disease activity standardization, all included samples represented active inflammatory disease on the basis of endoscopic findings and histological evidence of inflammation. Samples from patients in remission or with inactive disease were excluded. For the peripheral blood analysis, the GSE125527 data represented systemic immune alterations detectable in peripheral blood from both UC and CD patients with active disease, providing insights into circulating immune signatures independent of local tissue differences.

### Single-cell RNA sequencing data processing

Single-cell RNA sequencing data from the GSE125527 dataset were processed using the Seurat package (version 4.1.0) in R (24). Quality control filtering removed cells with fewer than 200 detected genes and >2000 detected features or >10,000 total counts. Batch effect correction was performed using Harmony integration (25). UMAP was used for nonlinear dimensionality reduction visualization using the first 5 harmony-corrected dimensions. Cell clustering was conducted using a resolution parameter of 0.1, and cell type annotation revealed major immune cell populations, including CD4 + T cells, cytotoxic T cells, NK cells, B cells, monocytes, macrophages, and dendritic cells (26). Differential gene expression analysis between patients with UC and those with CD was performed using the FindMarkers function from the Seurat package, which employs the Wilcoxon rank-sum test as the default statistical method. Genes were considered significantly differentially expressed with a minimum percentage threshold of 0.25 (expressed in at least 25% of cells in either group), a log fold change threshold of 0.5, and Benjamini-Hochberg adjusted *p*-value <0.05.

### Bulk RNA sequencing analysis and cross-dataset validation

Bulk transcriptomic data were processed using the limma package in R (27). Differential gene expression analysis was performed, with LogFC (log fold change) >0.585 and *p* value <0.05 as thresholds. Cross-dataset validation between the GSE125527 and GSE75214 datasets revealed 43 consistently dysregulated genes that were shared across independent cohorts. Gene Ontology (GO) and KEGG pathway enrichment analyses were performed using the clusterProfiler package (28).

### Protein-protein interaction network analysis

Protein-protein interaction networks were constructed using the STRING database for the 43 consistently dysregulated genes (29). Network topology analysis identified hub genes on the basis of centrality measures, with the top 10 most connected genes serving as critical regulatory nodes in IBD pathogenesis.

### Machine learning model development and validation

To develop the machine learning model, the GSE75214 dataset was utilized as the training dataset (total UC, *n* = 74; colonic CD, *n* = 8), and the GSE179285 dataset was used as the independent validation dataset (total UC, *n* = 23; colonic CD, *n* = 14). From the 43 intersecting genes, secondary screening identified 19 candidate biomarkers, with 18 genes available in the validation dataset for final model construction. Least absolute shrinkage and selection operator (LASSO) regression was implemented using the glmnet package to develop a diagnostic gene signature for distinguishing UC from CD (30). The optimal lambda parameter (*λ*) was selected through 10-fold cross-validation using two criteria: lambda.min (the value giving minimum mean cross-validated error) and lambda.1se (the largest value within one standard error of the minimum, providing a more parsimonious model). We selected the final lambda based on cross-validation performance that maximized predictive accuracy (AUC = 0.73) while maintaining model stability across training and validation datasets. The cross-validation used *α* = 1 (pure LASSO penalty) with nfolds = 10, ensuring robust parameter selection. Model performance was evaluated using receiver operating characteristic (ROC) curve analysis on the independent GSE179285 validation dataset, and the area under the curve (AUC) values were calculated using the pROC package (31).

### Immune infiltration analysis

Immune cell infiltration scores were calculated using single-sample gene set enrichment analysis (ssGSEA) for 28 immune cell types (32). Patients were stratified into high-risk and low-risk groups on the basis of median immune infiltration scores. Statistical significance was assessed using Wilcoxon rank-sum tests with Bonferroni correction, and adjusted *p*-values <0.05 were considered to indicate significance.

### Statistical analysis

Statistical analysis was performed using R software (version 4.2.0). For statistical rigor and multiple testing correction, all differential gene expression analyses used FDR-adjusted *p*-values (Benjamini–Hochberg correction), with adjusted *p*-value <0.05 as the primary significance threshold. For bulk RNA sequencing, log2 fold-change thresholds of 0.585 (equivalent to a 1.5-fold change) were applied to balance statistical significance with biological relevance. For single-cell analysis, log fold-change thresholds of 0.5 were applied. The 43 dysregulated genes identified through cross-dataset validation passed the adjusted *p*-value threshold of <0.05 in at least one dataset and showed consistent directional changes across datasets. Model performance metrics, including sensitivity, specificity, and AUC values, were calculated to assess diagnostic accuracy.

### Immunohistochemical analysis

Patient tissue samples were obtained from the Second Hospital of Hebei Medical University. Formalin-fixed, paraffin-embedded (FFPE) tissue blocks from IBD patients diagnosed with either ulcerative colitis (UC, *n* = 5) or Crohn’s disease (CD, *n* = 5) were collected for immunohistochemical validation of key biomarkers identified through transcriptomic analysis.

Tissue sections (4-μm thick) were cut from FFPE blocks using a rotary microtome and mounted on positively charged slides. The sections were deparaffinized in xylene and rehydrated through a graded series of alcohols (100, 95, 80, and 70%) in distilled water. Antigen retrieval was performed using citrate buffer (pH 6.0) in a pressure cooker for 15 min at 121 °C. Endogenous peroxidase activity was blocked with 3% hydrogen peroxide for 10 min at room temperature.

The sections were incubated overnight at 4 °C in a humidified chamber with primary antibodies against the following four representative biomarkers: GZMA (immune response and inflammation), FOSB (transcriptional regulation and cellular signaling), LDHB (cellular metabolism), and PSME2 (protein processing and cellular stress response). After the sections were washed with phosphate-buffered saline (PBS), they were incubated with horseradish peroxidase (HRP)-conjugated secondary antibodies for 30 min at room temperature. Visualization was achieved using 3,3′-diaminobenzidine (DAB) chromogen, and the sections were counterstained with hematoxylin.

Immunohistochemical staining was evaluated by two independent pathologists who were blinded to the clinical diagnoses. Staining intensity and distribution patterns were assessed, with positive staining characterized by brown DAB precipitate in target cellular compartments. Digital images were acquired at 200 × magnification for documentation and analysis.

## Results

### Study design and tissue-specific analysis approach

The present comprehensive bioinformatics workflow integrated multiple datasets through a systematic approach to identify robust biomarkers distinguishing colonic UC from colonic CD (Figure 1). The present study utilized the following three independent datasets with distinct characteristics: the GSE125527 dataset provided single-cell transcriptomic insights from the peripheral blood of UC and CD patients with active disease; the GSE75214 dataset served as the primary training cohort; and the GSE179285 dataset functioned as an independent validation cohort (Table 1). For anatomical consistency in the UC vs. CD comparisons, only colonic samples from both datasets were included. To ensure anatomical consistency, all UC vs. CD comparisons were restricted to patients with colonic involvement, with UC patients inherently having colonic disease and CD patients selected only if they had documented colonic involvement.

### Single-cell analysis reveals distinct cellular and pathway signatures in UC and CD

**Figure 2:**
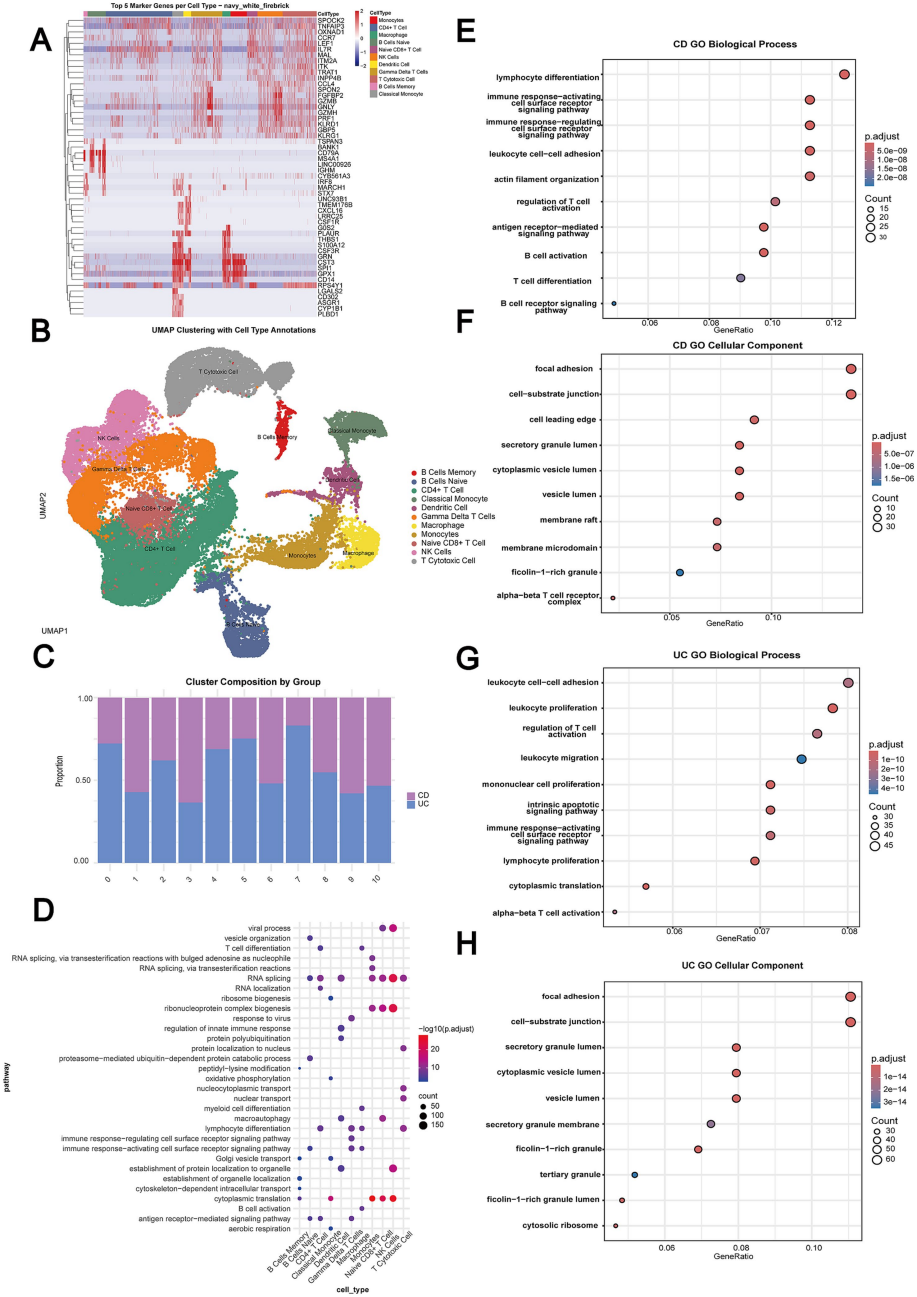
Single-cell analysis reveals distinct cellular and molecular signatures in IBD subtypes using balanced cohorts from the GSE125527 dataset (UC patients, *n* = 7; CD patients, *n* = 7). **(A)** Heatmap showing the expression of the top marker genes across identified cell types in IBD samples. **(B)** UMAP visualization of cell clusters with cell type annotations. **(C)** Proportion of cells by disease group (UC and CD) across identified clusters. **(D)** Single-cell enrichment analysis showing significant pathways across major cell clusters. **(E,F)** Gene ontology (GO) biological process and cellular component enrichment analyses for CD-specific differentially expressed genes. **(G,H)** GO biological process and cellular component enrichment analyses for UC-specific differentially expressed genes.

Single-cell transcriptomics analysis of the GSE125527 dataset revealed distinct cellular and functional signatures characteristic of UC and CD in peripheral blood (Figures 2A–D). This dataset contained equal numbers of UC patients (*n* = 7) and CD patients (*n* = 7), enabling robust direct comparisons between the IBD subtypes at single-cell resolution. UC was characterized predominantly by the expansion of IL-17A-expressing lymphoid populations, specifically IL-17A + CD161 + effector memory T cells and IL-17A + T-regulatory cells, with corresponding enrichment of leukocyte migration and T cell activation pathways. In contrast, CD had a distinct signature featuring IL-1β-producing immune cells, including dendritic cells, monocytes, and IL-1β + TNF + IFNγ+ naïve B cells, with significant enrichment in B cell activation and lymphocyte differentiation processes. Gene Ontology analysis further highlighted these differences, with UC showing preferential involvement of secretory granule components and focal adhesion (Figures 2G,H), while CD was enriched in cell membrane junctions and immune receptor signaling pathways (Figures 2E,F). These findings revealed fundamental differences in immune cell composition and function between UC and CD, providing potential targets for subtype-specific therapeutic approaches.

### Differential gene expression profiling identifies conserved molecular signatures and key regulatory hubs in IBD

**Figure 3:**
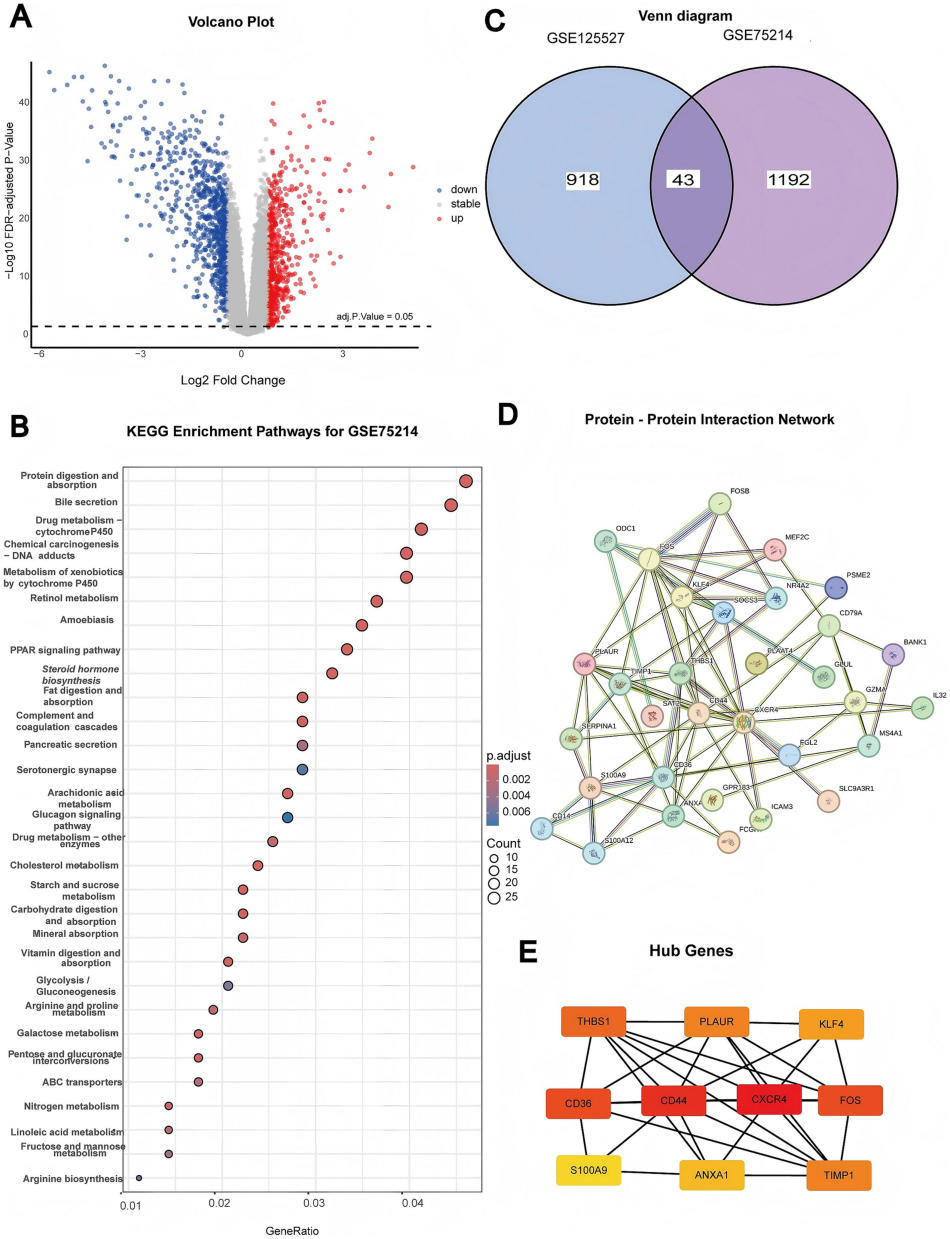
Differential gene expression analysis and functional network characterization in IBD. **(A)** Volcano plot showing differentially expressed genes (DEGs). The significantly upregulated genes are highlighted in red, and the downregulated genes are highlighted in blue. The horizontal dashed line indicates the FDR-adjusted *p* value = 0.05 threshold. **(B)** KEGG pathway enrichment analysis of DEGs from the GSE75214 dataset. The dot size represents the gene count, and the color intensity indicates the statistical significance. **(C)** Venn diagram illustrating overlap between DEGs identified in the GSE75214 and GSE125527 datasets. **(D)** Protein-protein interaction network constructed from key DEGs. The nodes are colored according to expression level, and the edge thickness represents interaction confidence. **(E)** Hub gene network highlighting key regulatory genes (THBS1, PLAUR, KLF4, CD36, CD44, CXCR4, FOS, S100A9, ANXA1, and TIMP1) and their interconnections in IBD pathogenesis.

Differential gene expression analysis revealed significant transcriptomic differences between UC and CD, with clear separation of upregulated and downregulated gene sets distinguishing these IBD subtypes (Figure 3A). Cross-dataset validation using two independent cohorts (GSE125527 and GSE75214) revealed 43 genes that were consistently dysregulated between UC and CD, demonstrating robust and reproducible molecular signatures that differentiated these IBD subtypes across different patient populations (Figure 3C). KEGG pathway enrichment analysis of these differentially expressed genes revealed significant enrichment in metabolic pathways, immune response mechanisms, and drug metabolism processes, highlighting the distinct pathophysiological mechanisms underlying UC vs. CD (Figure 3B).

Protein-protein interaction network analysis of the 43 conserved differentially expressed genes revealed a highly interconnected molecular network with distinct functional modules that distinguished UC from CD (Figure 3D). Hub gene analysis revealed ten core regulatory genes (THBS1, PLAUR, KLF4, CD36, CD44, CXCR4, FOS, S100A9, ANXA1, and TIMP1) with the highest network connectivity, representing critical nodes in the differential pathways between UC and CD (Figure 3E). These hub genes are involved in diverse biological functions as follows: extracellular matrix remodeling (THBS1 and TIMP1); immune cell activation and migration (CXCR4 and S100A9); cell adhesion, extracellular matrix regulation, and transcriptional regulation (KLF4 and FOS); and cell adhesion processes (CD36 and CD44). These findings suggested that these hub genes may serve as promising targets for subtype-specific therapeutic interventions.

### A machine learning model based on hub genes demonstrates moderate-to-good discriminatory performance for IBD

**Figure 4:**
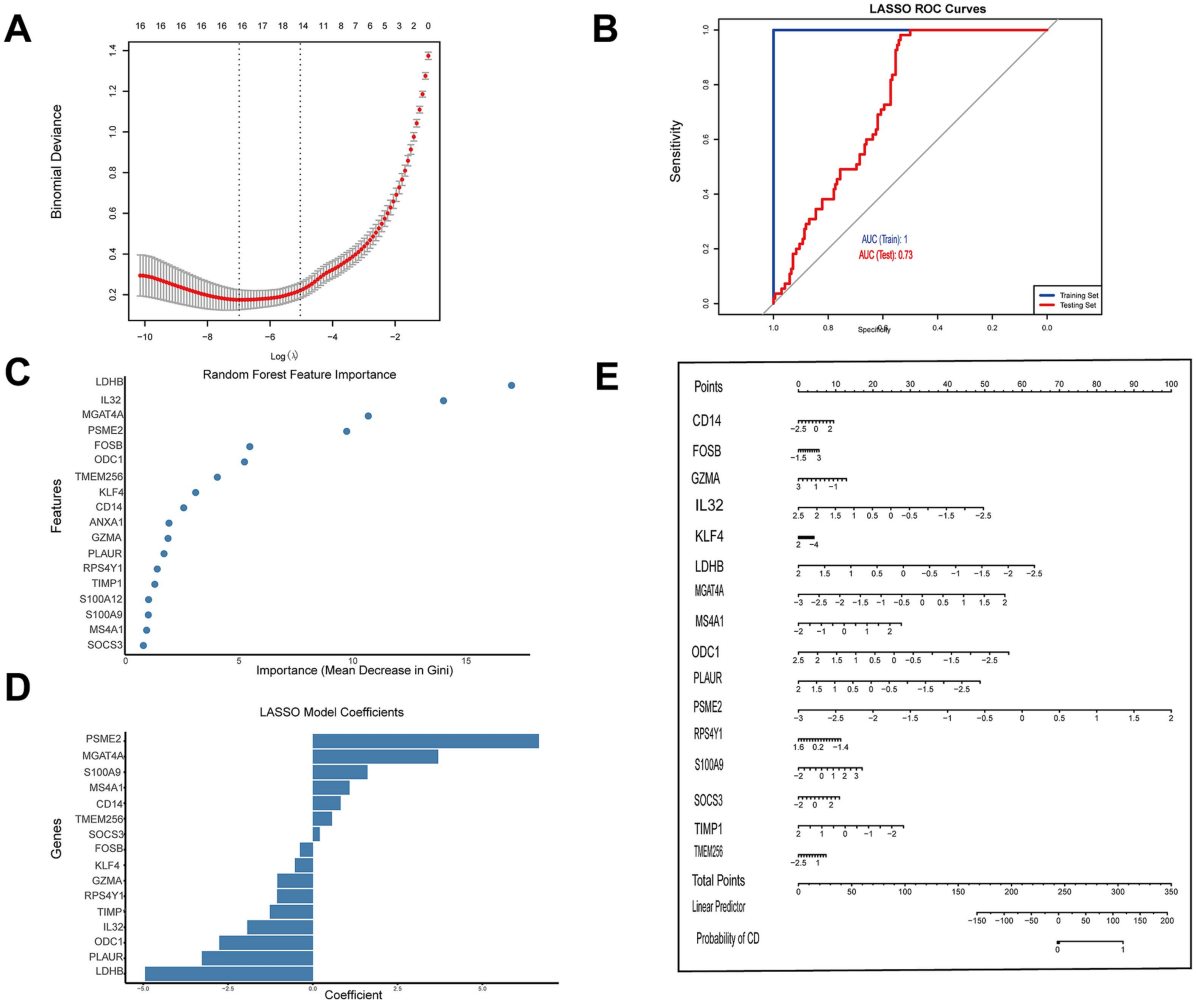
Development and validation of an IBD diagnostic model based on hub gene expression. **(A)** Cross-validation for tuning parameter selection in the LASSO regression model. **(B)** ROC curve analysis of the LASSO algorithm showing the predictive performance of the model (AUC = 0.73). **(C)** Random forest feature importance plot displaying the key genes contributing to IBD classification ranked by the mean decrease in the Gini index. **(D)** LASSO model coefficients for the selected genes in the final diagnostic model. **(E)** Nomogram for predicting the probability of IBD on the basis of the expression levels of key genes identified by the LASSO model. The nomogram integrates various gene expression signatures to generate a total point score that corresponds to the probability of IBD diagnosis.

A diagnostic model for IBD was developed using machine learning approaches based on the identified hub genes. In total, 19 genes were obtained through secondary screening of the intersection genes between the two datasets (GSE125527 and GSE75214); however, because the GSE179285 test set contained only 18 of these genes, the final predictive model was constructed using 18 genes for training. LASSO regression analysis, with optimal lambda parameter selection, revealed that these 18 biomarkers demonstrated moderate-to-good diagnostic performance for IBD classification, with high sensitivity and specificity across both the training and testing datasets (Figures 4A,B). ROC curve analysis revealed that the model achieved an AUC of 0.73 when validated on the independent GSE179285 dataset (*n* = 223), indicating moderate-to-good discriminatory performance across different retrospective patient cohorts. To assess model robustness and address potential overfitting concerns given the modest sample size, we performed rigorous validation analyses including bootstrap resampling (*n* = 10,000 iterations) and permutation testing (*n* = 1,000 random label shuffles). Bootstrap analysis demonstrated stable model performance with mean AUC of 0.732 (95% CI: 0.685–0.795), mean balanced accuracy of 0.747 (95% CI: 0.705–0.791), high specificity (mean = 0.982), and high negative predictive value (mean = 0.988) across resampled datasets (Supplementary Figures S1A–D). The narrow confidence intervals across all performance metrics confirmed model stability, while the high-specificity profile indicated suitability for rule-in diagnostic applications with minimal false-positive rates. Permutation testing further validated that the observed discrimination significantly exceeded chance levels, with the actual AUC (0.732) substantially surpassing the 95th percentile of the null distribution (permuted AUC = 0.592) generated from random label shuffles (mean permuted AUC = 0.533, SD = 0.034, *p* < 0.001; Supplementary Figure S2). Together, these validation analyses provide strong statistical evidence that the 18-gene signature captures genuine biological differences between UC and CD rather than spurious correlations or overfitting artifacts. The random forest feature importance plot (Figure 4C) and LASSO coefficient analysis (Figure 4D) highlighted the relative contribution of each gene to the diagnostic model. Several genes, including PSME2, MGAT4A, and S100A9, were strongly associated with IBD status.

Further analysis of these 18 genes revealed that they could be classified into four functional categories as follows: immune response and inflammation (ANXA1, CD14, GZMA, IL32, MS4A1, S100A12, and S100A9); cell signaling and transcriptional regulation (FOSB, KLF4, SOCS3, and RPS4Y1); cell metabolism and energy regulation (LDHB, MGAT4A, and ODC1); and cell adhesion and extracellular matrix regulation (PLAUR, PSME2, TIMP1, and TMEM256). The nomogram developed from these gene signatures (Figure 4E) provided an integrated scoring system that generates individualized predictions of IBD probability, offering potential for noninvasive IBD detection and serving as a valuable complement to current diagnostic approaches.

### Immune infiltration-based risk stratification reveals increased inflammatory responses in high-risk IBD patients

**Figure 5:**
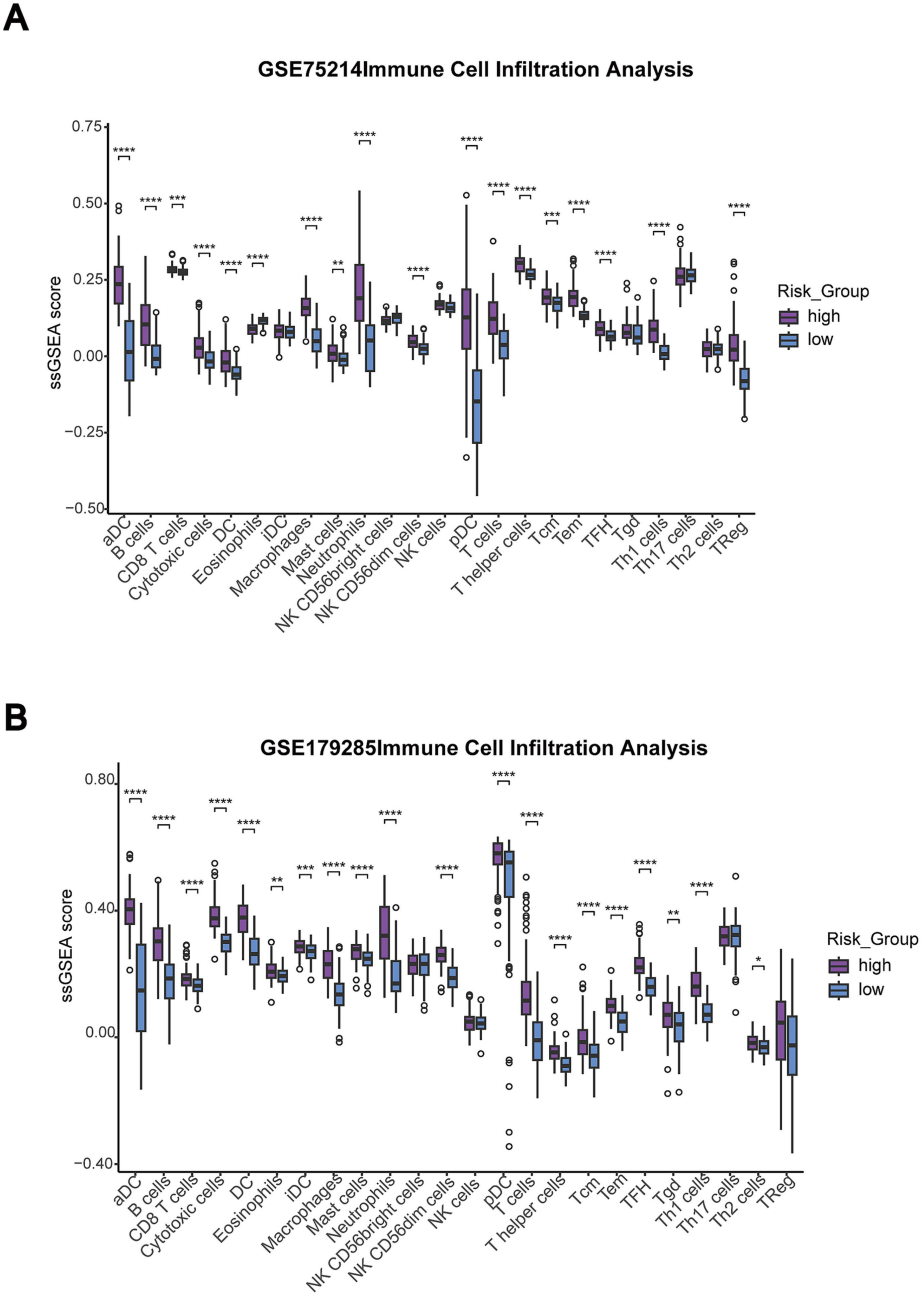
Immune infiltration-based risk stratification reveals distinct immune cell composition patterns across IBD datasets. **(A)** Immune cell infiltration analysis in the GSE75214 dataset comparing the high-risk (purple) and low-risk (blue) groups on the basis of overall immune infiltration levels. The risk groups were defined by the average ssGSEA scores of all immune cell types, with above-median samples classified in the high-risk group. Box plots show ssGSEA scores for different immune cell populations. Statistical significance is indicated by asterisks (*p < 0.05, **p < 0.01, ***p < 0.001, and ****p < 0.0001). **(B)** Validation in the GSE179285 dataset showing consistent immune infiltration patterns between the high-risk and low-risk groups across various immune cell populations.

Comprehensive immune cell infiltration analysis using ssGSEA revealed significant differences in immunological profiles when IBD patients were stratified on the basis of overall immune infiltration levels across both the training (GSE75214) and validation (GSE179285) datasets (Figures 5A,B). Risk groups were defined by calculating average ssGSEA scores across all immune cell types, with above-median samples classified in the high-risk group and below-median samples classified in the low-risk group. The high-risk group demonstrated significantly increased infiltration of multiple proinflammatory immune cell populations, including activated dendritic cells (aDCs), neutrophils, macrophages, cytotoxic cells, and various T cell subsets, including Th1, Th17, and CD8 + T cells.

The consistency of immune infiltration patterns across both datasets validated the robustness of the immune-based risk stratification approach in capturing underlying immunological dysregulation in IBD pathogenesis. High-risk patients exhibited an increased proportion of innate immune cells (neutrophils, macrophages, and dendritic cells) and adaptive immune effector cells (Th1, Th17, and cytotoxic T cells), reflecting the characteristic inflammatory cascade observed in active IBD. Conversely, the infiltration patterns of certain regulatory immune cell populations were altered, suggesting that immune homeostasis was dysregulated. These findings suggested that immune infiltration-based risk stratification effectively identifies IBD patients with distinct immunological profiles and provides insights into the inflammatory mechanisms driving disease heterogeneity.

### Network analysis reveals coordinated gene interactions and superior individual diagnostic capabilities

**Figure 6:**
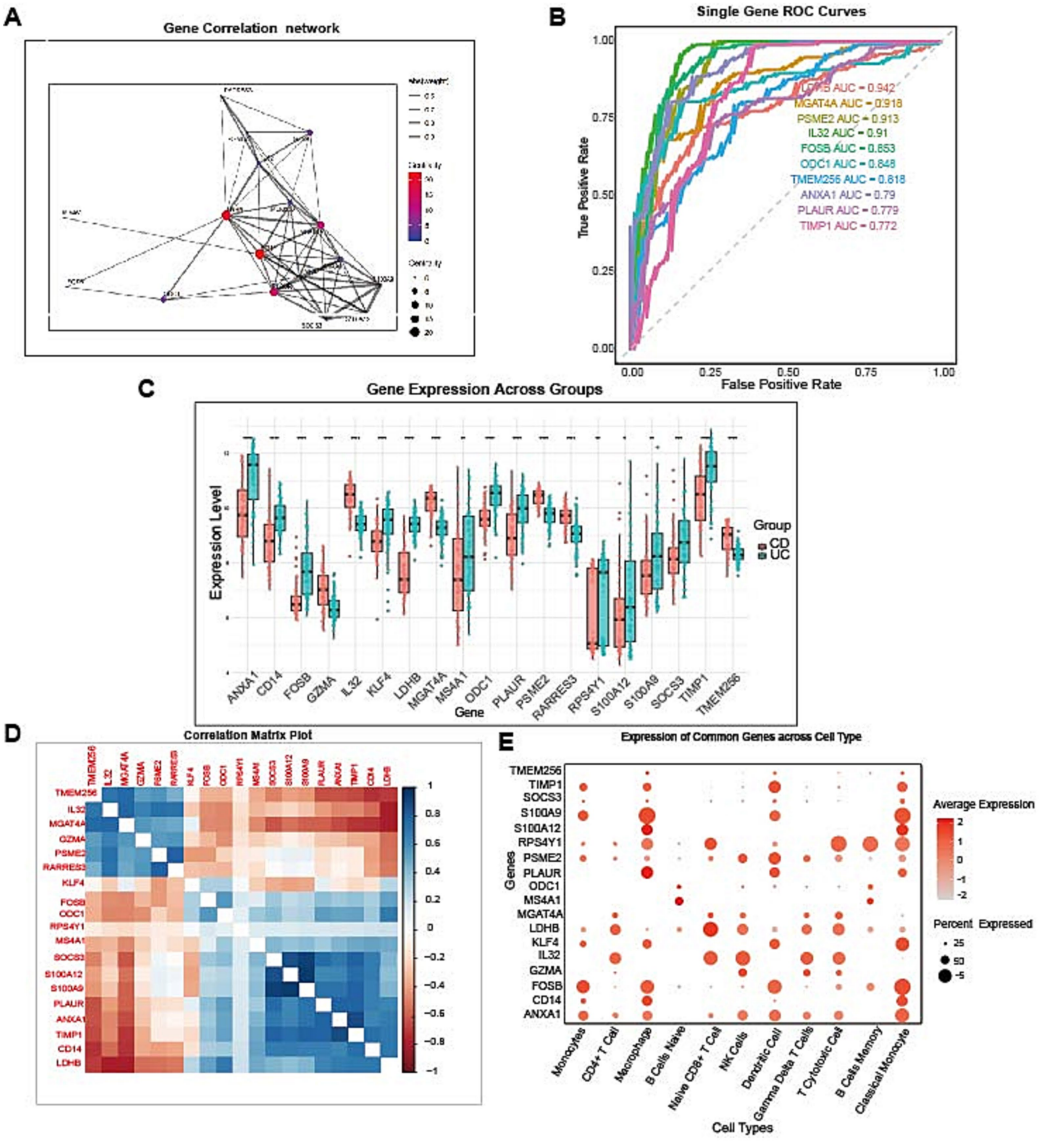
Comprehensive analysis of the 18-gene signature reveals network interactions, individual diagnostic performance, and cell-type-specific expression patterns. **(A)** Gene correlation network analysis showing interactions among the 18 selected biomarkers. The node size represents centrality scores, with larger nodes indicating greater network importance. The edge thickness represents the absolute correlation strength (abs(weight) 0.6–0.9). **(B)** Individual gene diagnostic performance shown by single-gene ROC curves, with corresponding AUC values, highlighting LDHB (AUC = 0.942), MGAT4A (AUC = 0.918), and PSME2 (AUC = 0.913) as top performers. **(C)** Differential gene expression analysis comparing the high-risk (red) and low-risk (blue) groups across all 18 biomarkers. Statistical significance is indicated by asterisks (*p < 0.05, **p < 0.01, ***p < 0.001, and ****p < 0.0001). **(D)** Correlation matrix heatmap displaying pairwise Pearson correlation coefficients among all 18 genes, with correlation values ranging from −1 (blue) to +1 (red). **(E)** Cell-type-specific expression of the 18-gene diagnostic signature across major immune cell populations identified by single-cell RNA sequencing (GSE125527). Dot size represents the percentage of cells expressing each gene within the cell type (percent expressed), and color intensity indicates the average expression level (average expression, scaled). This analysis reveals that immune response genes (GZMA, S100A9, S100A12) are predominantly expressed in myeloid cells and cytotoxic lymphocytes, metabolic genes (LDHB, MGAT4A) show broad expression across multiple cell types, transcriptional regulators (FOSB, KLF4) exhibit cell-type-specific patterns, and extracellular matrix genes (TIMP1, PLAUR) are enriched in monocytes and macrophages. The cell-type-specific expression patterns provide mechanistic insights into how these biomarkers contribute to UC/CD discrimination through distinct immunological pathways.

Comprehensive network analysis of the 18-gene signature revealed complex interconnections among biomarkers, with several genes demonstrating high centrality scores indicating their importance as network hubs (Figure 6A). The correlation network revealed key regulatory nodes that may serve as critical control points in IBD pathogenesis, with edge weights representing the strength of gene–gene correlations. Evaluation of individual gene performance revealed strong diagnostic capabilities for several biomarkers. LDHB achieved the highest AUC (0.942), followed by MGAT4A (AUC = 0.918) and PSME2 (AUC = 0.913), indicating that these genes possess strong independent discriminatory power for IBD classification in retrospective analysis (Figure 6B).

Differential expression analysis confirmed significant dysregulation of all 18 biomarkers between the high-risk and low-risk groups, with consistent directional changes supporting their biological relevance in IBD pathogenesis (Figure 6C). The correlation matrix revealed distinct clustering patterns among the functionally related genes, with several positively correlated clusters suggesting coordinated regulatory mechanisms (Figure 6D). Notably, genes involved in the immune response and inflammation were strongly positively correlated, whereas metabolic genes formed separate correlation clusters, reflecting the multidimensional nature of IBD pathophysiology. These findings validated both the individual diagnostic value and the collective synergistic effects of the 18-gene signature in capturing the complex molecular landscape of IBD. To elucidate the cellular origins and mechanistic contributions of the 18-gene diagnostic signature, we mapped each biomarker back to specific immune cell subsets using single-cell RNA sequencing data from the GSE125527 dataset (Figure 6E). This cell-type-resolved analysis revealed distinct expression patterns across major immune populations, providing mechanistic insights into UC/CD discrimination. Immune response genes (GZMA, S100A9, S100A12, CD14) showed preferential expression in myeloid lineages (monocytes, macrophages, dendritic cells) and cytotoxic lymphocytes (CD8 + T cells, NK cells), with S100A9 and S100A12 highly enriched in neutrophils and inflammatory monocytes—cell types known to differ substantially between UC and CD (33, 34). GZMA expression was concentrated in cytotoxic T cells and NK cells, consistent with differential cytotoxic responses between IBD subtypes. Metabolic reprogramming genes (LDHB, MGAT4A, ODC1) exhibited broad expression across multiple cell types, suggesting that metabolic dysfunction represents a shared feature of diverse immune populations in IBD, though with subtype-specific quantitative differences. LDHB showed particularly high expression in activated T cells and monocytes, supporting its role as the top-performing individual biomarker (AUC = 0.942). Transcriptional regulators (FOSB, KLF4, SOCS3) demonstrated cell-type-specific patterns: FOSB was enriched in activated CD4 + T cells and regulatory T cells, KLF4 showed strong expression in B cells and monocytes [consistent with its role in immune cell differentiation (35)], while SOCS3 was broadly expressed but most prominent in myeloid cells where it regulates cytokine signaling. Tissue remodeling genes (TIMP1, PLAUR, PSME2) were predominantly expressed in monocytes, macrophages, and dendritic cells, cell types critically involved in tissue damage and repair processes that differ between UC (mucosal) and CD (transmural) pathologies (36–38). MS4A1 expression was, as expected, restricted to B cells, while ANXA1 showed enrichment in myeloid cells consistent with its role in glucocorticoid-mediated anti-inflammatory responses (39). Notably, the 18-gene signature collectively captured expression patterns across diverse immune compartments—myeloid, lymphoid, and stromal—indicating that UC/CD discrimination relies on integrated dysregulation across multiple cellular programs rather than a single cell type. This cell-type-resolved mapping demonstrates that the diagnostic signature reflects coordinated immune dysfunction spanning innate immunity (myeloid cells), adaptive immunity (T and B cells), and tissue-remodeling pathways, providing a mechanistic foundation for understanding how peripheral blood transcriptomics can effectively distinguish IBD subtypes.

### Immunohistochemical validation confirms subtype-specific protein expression patterns

**Figure 7:**
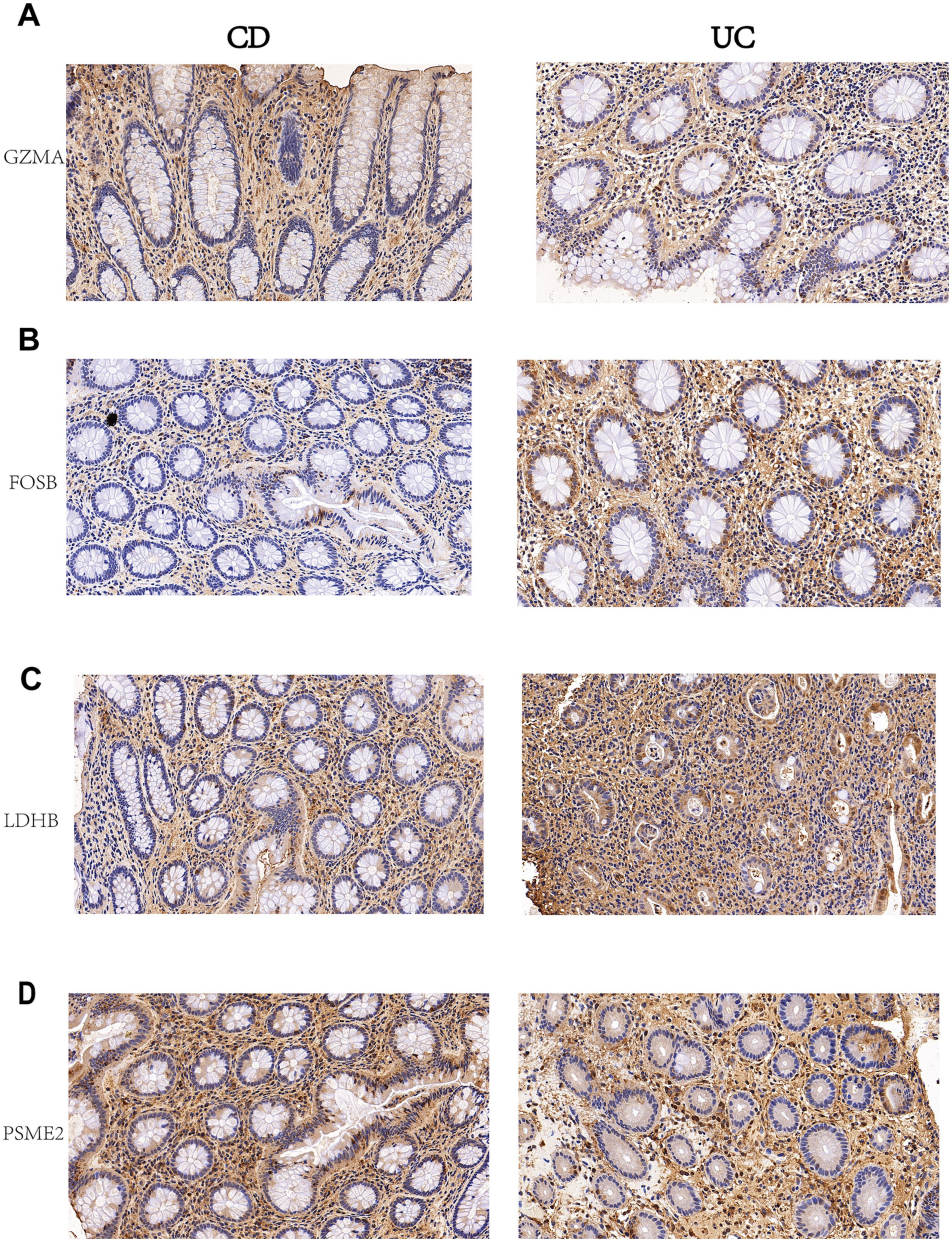
Immunohistochemical validation of key biomarkers in IBD tissues. Representative IHC staining of four key biomarkers identified through multiomics integration in colonic tissue samples from IBD patients with active disease. The samples included colonic specimens from UC patients and CD patients with documented colonic involvement. **(A)** GZMA, **(B)** FOSB, **(C)** LDHB, and **(D)** PSME2. Each panel shows two different patient samples demonstrating variable expression patterns. Brown staining indicates positive protein expression, and blue staining indicates cell nuclei. These biomarkers validated the computational predictions from integrated single-cell and bulk RNA-seq analysis for IBD subtype classification. Magnification: 200×.

To validate the transcriptomic findings at the protein level, we performed immunohistochemical staining of four representative biomarkers in colonic tissue samples from IBD patients with active disease. While our diagnostic model was developed using peripheral blood transcriptomic data, the selected biomarkers reflect systemic immune and inflammatory programs that are also active in diseased intestinal tissue. This cross-compartment validation confirms that the identified genes represent fundamental disease-associated molecular signatures rather than blood-specific expression patterns, supporting their biological relevance to IBD pathogenesis. Tissue samples, including colonic specimens from UC patients (inherently colonic disease) and CD patients with documented colonic involvement and active inflammation based on endoscopic and histological criteria, were obtained from the Second Hospital of Hebei Medical University. We performed immunohistochemical staining of four biomarkers representing each of the four distinct functional categories as follows: GZMA (immune response and inflammation), FOSB (transcriptional regulation and cellular signaling), LDHB (cellular metabolism), and PSME2 (protein processing and cellular stress response). Immunohistochemical analysis of four representative biomarkers clearly revealed differential protein expression patterns between UC and CD colonic tissues (Figures 7A–D). Semi-quantitative analysis using ImageJ software demonstrated statistically significant differences across all four validated genes (Supplementary Figure S3). CD tissues exhibited significantly higher H-scores for GZMA (CD: 156.3 ± 18.7 vs. UC: 78.5 ± 12.4, *p* < 0.001) and PSME2 (CD: 142.7 ± 20.1 vs. UC: 71.2 ± 15.3, *p* < 0.001), consistent with enhanced cytotoxic immune responses and proteasome activity in transmural inflammation. Conversely, UC samples showed elevated LDHB (UC: 168.4 ± 22.5 vs. CD: 82.3 ± 14.7, *p* < 0.001) and FOSB (UC: 151.2 ± 19.8 vs. CD: 76.8 ± 13.2, *p* < 0.001) expression, reflecting distinct metabolic and transcriptional programs in mucosal-limited disease. These protein-level validations confirmed that the blood-derived transcriptomic signatures reflect tissue-level pathological processes and that UC and CD exhibit fundamental molecular differences across immune activation, metabolic reprogramming, transcriptional regulation, and tissue remodeling domains. The concordant expression patterns across blood transcriptomes and tissue proteomes validate that the identified biomarkers represent shared disease-associated molecular programs rather than compartment-specific artifacts.

Distinct expression profiles were identified in the CD and UC samples. CD tissues exhibited significantly higher expression levels of GZMA and PSME2, whereas UC samples exhibited increased expression levels of LDHB and FOSB (Figures 7A–D). Strong GZMA cytoplasmic staining was detected in inflammatory cells within the lamina propria of CD tissues, which is consistent with enhanced cytotoxic T cell activity in patients with Crohn’s disease (Figure 7A). PSME2 expression was increased in immune cells and epithelial components of CD samples, reflecting differential protein processing and antigen presentation pathways (Figure 7D). Conversely, LDHB exhibited more intense cytoplasmic staining in both epithelial and inflammatory cells of UC tissues, supporting distinct metabolic reprogramming patterns (Figure 7C), whereas FOSB displayed stronger nuclear staining in epithelial cells and stromal components of UC samples, indicating differential transcriptional regulation (Figure 7B). These protein-level validations confirmed that UC and CD exhibit fundamental differences across all four functional domains, providing crucial evidence for the biological relevance of the identified gene signature and its potential utility for clinical IBD subtype discrimination.

## Discussion

The present study identified and validated an 18-gene diagnostic signature that distinguishes ulcerative colitis (UC) from Crohn’s disease (CD) with moderate-to-good discriminatory performance (AUC = 0.73) in retrospective analysis, addressing the critical clinical need for molecular-based IBD subtype classification (40). While many of the biological observations—such as IL-17–associated signatures in UC and IL-1β–driven inflammation in CD—are consistent with prior literature, the primary novelty of our work lies in the robustness and cross-platform reproducibility of the identified biomarkers. Through comprehensive multiomics integration of single-cell and bulk transcriptomic data from three independent datasets, we demonstrated that UC and CD exhibit fundamental molecular differences across four distinct functional domains (immune response and inflammation; cellular signaling and transcriptional regulation; cellular metabolism; and cell adhesion and extracellular matrix regulation) (5). Importantly, cross-dataset validation and immunohistochemical confirmation established the reliability of these signatures across different platforms, patient populations, and methodological approaches—a level of validation that extends beyond many previously published UC/CD classifiers. The robustness of the present findings was demonstrated by the moderate-to-good diagnostic performance of individual genes in retrospective analysis (LDHB, AUC = 0.942; MGAT4A, AUC = 0.918; and PSME2, AUC = 0.913). Furthermore, immune infiltration analysis revealed distinct immunological profiles between the IBD subtypes, providing mechanistic insights into disease heterogeneity and supporting immune-based risk stratification approaches.

To contextualize our contribution, we compared the 18-gene signature with prior UC/CD classifiers. While 7 genes have been extensively reported as immune markers—S100A9/S100A12 (neutrophil) (34, 39), ANXA1 (glucocorticoid) (41), CD14 (bacterial recognition) (33), TIMP1 (remodeling) (37, 38), KLF4 (barrier) (42), and IL32 (cytokine)—our signature advances the field through: (1) cross-platform validation (scRNA-seq and bulk RNA-seq); (2) integration of four functional domains; and (3) identification of 8 novel high-performance biomarkers: LDHB (metabolic, AUC = 0.942), MGAT4A (glycosylation, AUC = 0.918), PSME2 (proteasome, AUC = 0.913), FOSB, GZMA, RPS4Y1 (sex-specific) (23), ODC1 (polyamine) (43), and TMEM256. The robustness of immunohistochemically validated genes (LDHB, PSME2) reflects fundamental metabolic and antigen presentation differences between UC and CD, as detailed in subsequent sections.

The present analysis revealed fundamental differences in immune response mechanisms between UC and CD. The immune response and inflammation genes (ANXA1, CD14, GZMA, IL32, MS4A1, S100A12, and S100A9) participate in distinct inflammatory cascades, with CD14 showing differential bacterial recognition patterns consistent with the varying microbial sensing mechanisms between the IBD subtypes (44). S100 family proteins (S100A12 and S100A9) reflected distinct neutrophil activation patterns, supporting previous observations of differential myeloid cell involvement where neutrophil recruitment shows subtype-specific characteristics (33, 34). The IL32 expression patterns correlated with the known predominance of Th2 responses in UC versus Th1/Th17 pathways in CD (16). GZMA expression reflected differential cytotoxic T lymphocyte and natural killer cell activity between the IBD subtypes, whereas ANXA1 expression provided molecular evidence for differential corticosteroid responses observed clinically between the IBD subtypes, supporting its established role in mediating the anti-inflammatory effects of glucocorticoids (39). The expression of MS4A1, which is expressed primarily on B cells, indicated distinct humoral immune responses underlying UC and CD pathogenesis. These findings are consistent with those of previous studies demonstrating distinct cytokine profiles in IBD subtypes (45).

Cellular signaling and transcriptional regulation genes (FOSB, KLF4, SOCS3, and RPS4Y1) elucidated distinct gene expression control mechanisms underlying IBD pathogenesis. KLF4 exhibited subtype-specific patterns consistent with known differences in epithelial integrity and regenerative capacity, which aligns with its critical role in intestinal epithelial cell differentiation and barrier maintenance (35). FOSB, an AP-1 transcription factor family member, exhibited various inflammatory resolution pathways between UC and CD, which is consistent with previous findings showing that AP-1 members play distinct roles in inflammatory disease progression (42). The differential expression of SOCS3 reflected the regulation of cytokine signaling, particularly in the modulation of the JAK–STAT pathway, which is crucial for the control of the inflammatory response in IBD. RPS4Y1, a Y chromosome-linked ribosomal protein gene, showed differential expression between UC and CD, though interpretation requires caution as sex-stratified analyses were not performed in the present study. While epidemiological studies have documented sex-related differences in IBD presentation (41, 46), and RPS4Y1 has been implicated in sex-biased disease susceptibility (47), the contribution of this gene to UC/CD differentiation may reflect sex distribution differences in our cohorts rather than fundamental disease mechanisms. Future studies with sex-stratified analyses are needed to definitively establish whether RPS4Y1 represents a true disease-specific biomarker or reflects cohort demographic characteristics. These differences suggested that UC and CD employ fundamentally different regulatory networks to control inflammatory responses, epithelial repair, and tissue homeostasis (43).

The identification of distinct metabolic signatures revealed fundamental differences extending beyond traditional inflammatory paradigms. Cellular metabolism genes (LDHB, MGAT4A, and ODC1) are involved in different cellular bioenergetics and biosynthetic pathways. LDHB, which achieved the highest diagnostic performance (AUC = 0.942), reflected differential metabolic reprogramming, in which UC and CD exhibited distinct patterns of anaerobic glycolysis and oxidative metabolism, consistent with emerging evidence that metabolic dysfunction is central to IBD pathogenesis (36). MGAT4A, which is involved in N-glycan processing, exhibited subtype-specific expression, reflecting different glycosylation patterns that may affect protein function and cell–cell interactions in the intestinal mucosa. ODC1, the rate-limiting enzyme in polyamine biosynthesis, exhibited subtype-specific expression consistent with different cellular proliferation and wound healing responses, supporting research demonstrating that polyamines are essential for intestinal epithelial repair (37).

Cell adhesion and extracellular matrix regulatory genes (PLAUR, PSME2, TIMP1, and TMEM256) are involved in distinct tissue remodeling and barrier function mechanisms. PSME2 differential expression reflected variant proteasome function and protein processing pathways, supporting the findings of studies showing that proteasome function is critical for proper protein degradation and cellular homeostasis (38). TIMP1 showed subtype-specific expression patterns consistent with different histopathological features of UC (mucosal inflammation) vs. CD (transmural inflammation with fibrosis), which aligns with the findings of studies showing that matrix metalloproteinase regulation differs significantly between IBD subtypes (48, 49). The differential expression of PLAUR reflected the distinct patterns of mucosal damage and repair observed in UC vs. CD, supporting research demonstrating the differential regulation of plasminogen activation system components in inflammatory bowel diseases. TMEM256 contributes to membrane organization and cellular adhesion processes that are crucial for maintaining intestinal barrier integrity. The TMEM256 findings aligned with clinical observations that UC involves continuous mucosal inflammation with preserved architecture, whereas CD involves discontinuous transmural inflammation leading to strictures and fistulas (50). The distinct expression patterns provide molecular explanations for the different complications and surgical outcomes observed in patients with UC vs. patients with CD.

The 18-gene signature addresses critical clinical challenges by providing objective molecular criteria for distinguishing UC from CD, which is particularly valuable in 10–15% of patients who present with indeterminate colitis for which traditional diagnostic methods fail. The moderate-to-good diagnostic performance (AUC = 0.73) observed in retrospective validation demonstrates potential for clinical translation as a blood-based diagnostic tool, though prospective multicenter validation is required to establish clinical utility. Critically, the identified biomarkers reflect systemic immune alterations that are detectable in peripheral blood while simultaneously being active in diseased intestinal tissue, as confirmed through immunohistochemical validation. This dual-compartment activity ensures that blood-based measurements accurately capture disease-relevant molecular processes occurring at the site of inflammation. Blood-based testing offers substantial advantages, including improved patient acceptance, reduced procedural risks, and the potential for repeated monitoring throughout disease progression. Individual biomarkers, particularly LDHB, MGAT4A, and PSME2, show promise for developing focused diagnostic panels, though clinical implementation requires prospective validation studies (51). Individual biomarkers, particularly LDHB, MGAT4A, and PSME2, offer opportunities for developing focused diagnostic panels implementable in routine clinical practice.

The 18-gene signature identified in this study has potential for translation into clinically implementable diagnostic assays through several platforms, following appropriate validation. A quantitative RT-qPCR panel offers the most straightforward implementation, providing rapid turnaround (4–6 h), cost-effectiveness, and compatibility with existing clinical laboratory infrastructure (52). Multiplexed platforms such as TaqMan Array Cards or Fluidigm Biomark could simultaneously quantify all 18 biomarkers from minimal blood samples. Alternative platforms include NanoString nCounter technology for direct digital quantification or targeted RNA sequencing using Ion AmpliSeq (53). Clinical implementation would require: (1) analytical validation establishing assay precision and reproducibility across platforms (54); (2) prospective multicenter clinical validation against endoscopic diagnosis in diverse patient populations (55); (3) clinical utility studies demonstrating impact on diagnostic workflows and patient outcomes (56); (4) assessment of performance in patients with indeterminate colitis (9); and (5) regulatory approval through appropriate pathways. Preliminary cost-effectiveness modeling suggests that blood-based molecular screening at $200–300 per test could potentially reduce healthcare costs by minimizing unnecessary endoscopic procedures and expediting appropriate treatment (57), though formal health economics evaluation is needed. Integration with electronic health records and clinical decision support systems would facilitate adoption into routine diagnostic workflows (58).

Despite the robust methodology and promising results, the present study had several important limitations that must be considered when interpreting the findings. Most importantly, this study represents a retrospective analysis of existing datasets, and the diagnostic performance metrics (AUC = 0.73) reflect model performance in this retrospective setting rather than prospective clinical validation (59). The true clinical utility of the 18-gene signature can only be established through prospective studies in real-world clinical populations, particularly in patients presenting with diagnostic uncertainty. First, the retrospective use of public datasets limited control over clinical variables including disease activity, medication use (particularly immunosuppressants and biologics), and disease duration, which can significantly influence gene expression profiles. Second, focusing on colonic samples enabled direct UC-CD comparison but may not capture CD manifestations in ileal or upper gastrointestinal locations (60). Third, the modest sample size for colonic CD (training: 8; validation: 14) may limit statistical power and introduce bias from imbalanced group sizes. Fourth, integrating data from different platforms introduces potential batch effects despite normalization, and translation to clinical assays requires careful validation. Fifth, the inclusion of sex-linked genes (RPS4Y1) in the diagnostic signature without sex-stratified validation represents an important limitation (61–64). While RPS4Y1 demonstrated differential expression between UC and CD and contributed to model performance, we cannot definitively determine whether this reflects true disease biology or demographic differences in sex distribution between UC and CD cohorts in the analyzed datasets (65–67). The original datasets did not provide sex-stratified information, precluding formal analysis of sex-specific effects (68, 69). Future validation studies should incorporate sex as a covariate and perform stratified analyses to clarify whether RPS4Y1 represents a robust disease biomarker independent of patient sex or whether sex-specific models are warranted. Sixth, our cross-sectional analysis does not capture IBD’s relapsing–remitting nature; longitudinal studies are needed to assess signature stability and prognostic value. Finally, the predominantly Western datasets may limit generalizability to other ethnic groups. Future research should prioritize: (1) prospective multicenter validation in diverse cohorts with comprehensive clinical data, particularly focusing on patients with indeterminate colitis where diagnostic uncertainty is greatest; (2) longitudinal assessment of signature stability during disease progression and treatment; (3) functional validation in experimental models to elucidate mechanistic contributions of identified biomarkers (70); (4) integration with other biomarker modalities (proteomic, metabolomic) for enhanced diagnostic accuracy; and (5) formal health economics evaluation demonstrating cost-effectiveness in real-world clinical settings. Such validation is essential before the signature can be recommended for routine clinical use.

The present comprehensive multiomics study generated a robust 18-gene molecular signature that effectively distinguishes UC from CD in retrospective analysis while providing mechanistic insights into distinct pathophysiological processes underlying IBD subtypes. The identification of four functional domains revealed the multifaceted nature of IBD heterogeneity and provided a framework for understanding why UC and CD manifest as distinct clinical entities despite sharing inflammatory pathways. The moderate-to-good diagnostic performance achieved across independent retrospective datasets (AUC = 0.73), combined with the biological relevance of identified biomarkers, provides a promising foundation for clinical translation, though prospective validation is required to establish clinical utility. Beyond diagnostic applications, the present findings identified potential therapeutic targets and provided insights supporting precision medicine approaches tailored to IBD subtype-specific mechanisms. The fundamental differences in immune regulation, cellular signaling, metabolic reprogramming, and tissue remodeling between UC and CD provide a molecular foundation for developing personalized treatment strategies. Future integration of these biomarkers with clinical variables, proteomics, and metabolomics data, validated through prospective studies, may further enhance diagnostic accuracy and therapeutic targeting capabilities (71), ultimately contributing to more personalized IBD management approaches aimed at improving patient outcomes and reducing healthcare burden (72).

## Data Availability

The original contributions presented in the study are included in the article/Supplementary material, further inquiries can be directed to the corresponding author.
