# Supplementary figures and images for: Multiomics integration of single-cell transcriptomics and bulk sequencing data identifies key biomarkers and predictive models for IBD subtype classification
Source: Front Med (Lausanne). 2026 Mar 19;13:1729642. doi: 10.3389/fmed.2026.1729642 (PMC13044067; doi:10.3389/fmed.2026.1729642)

S1


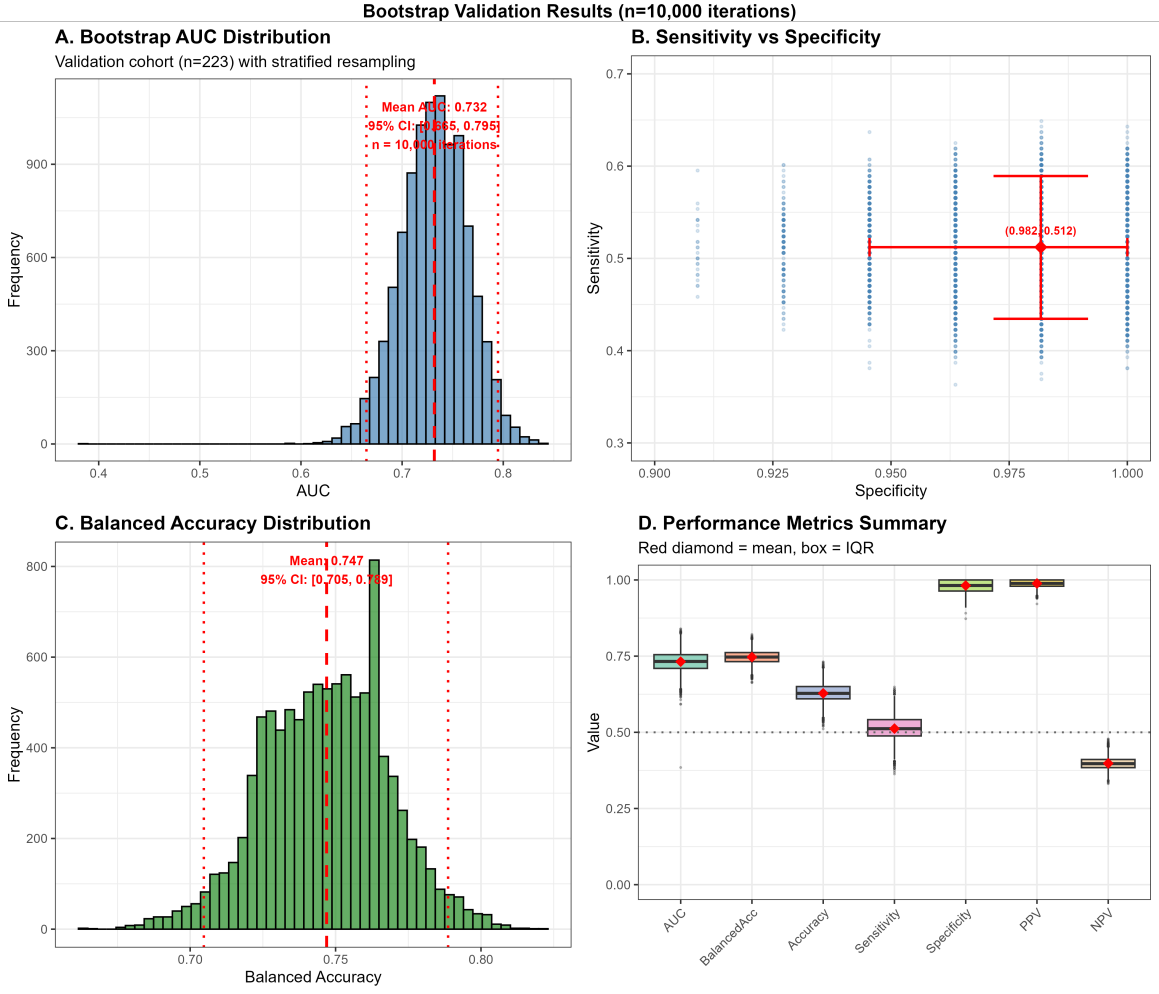


S2


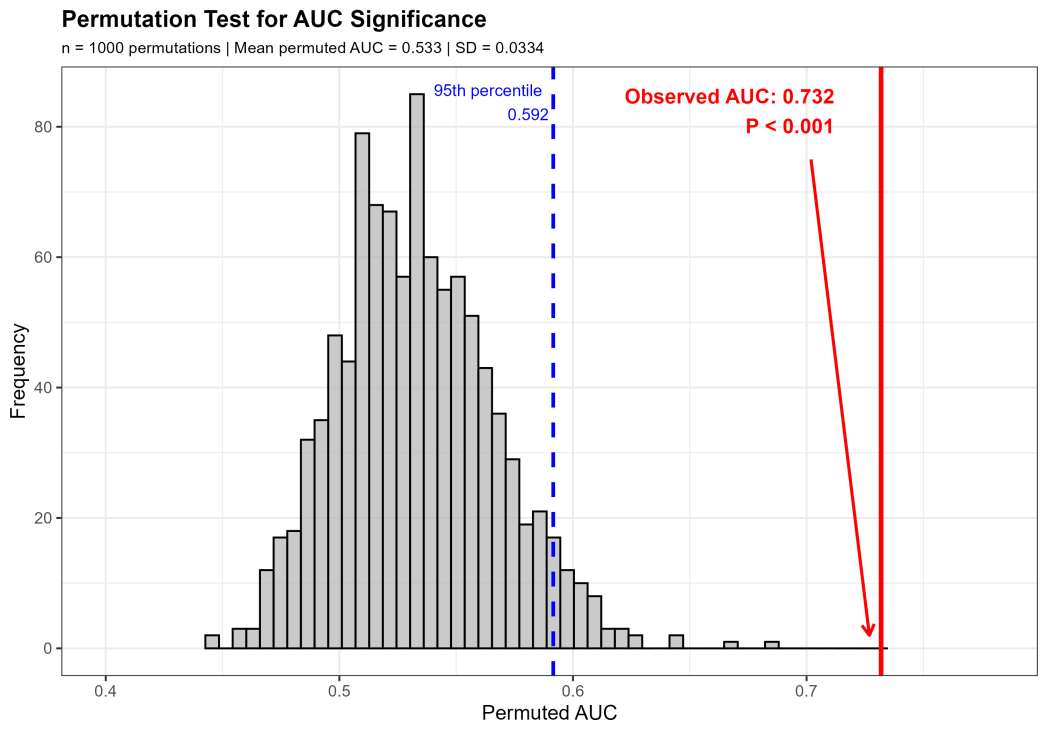


S3


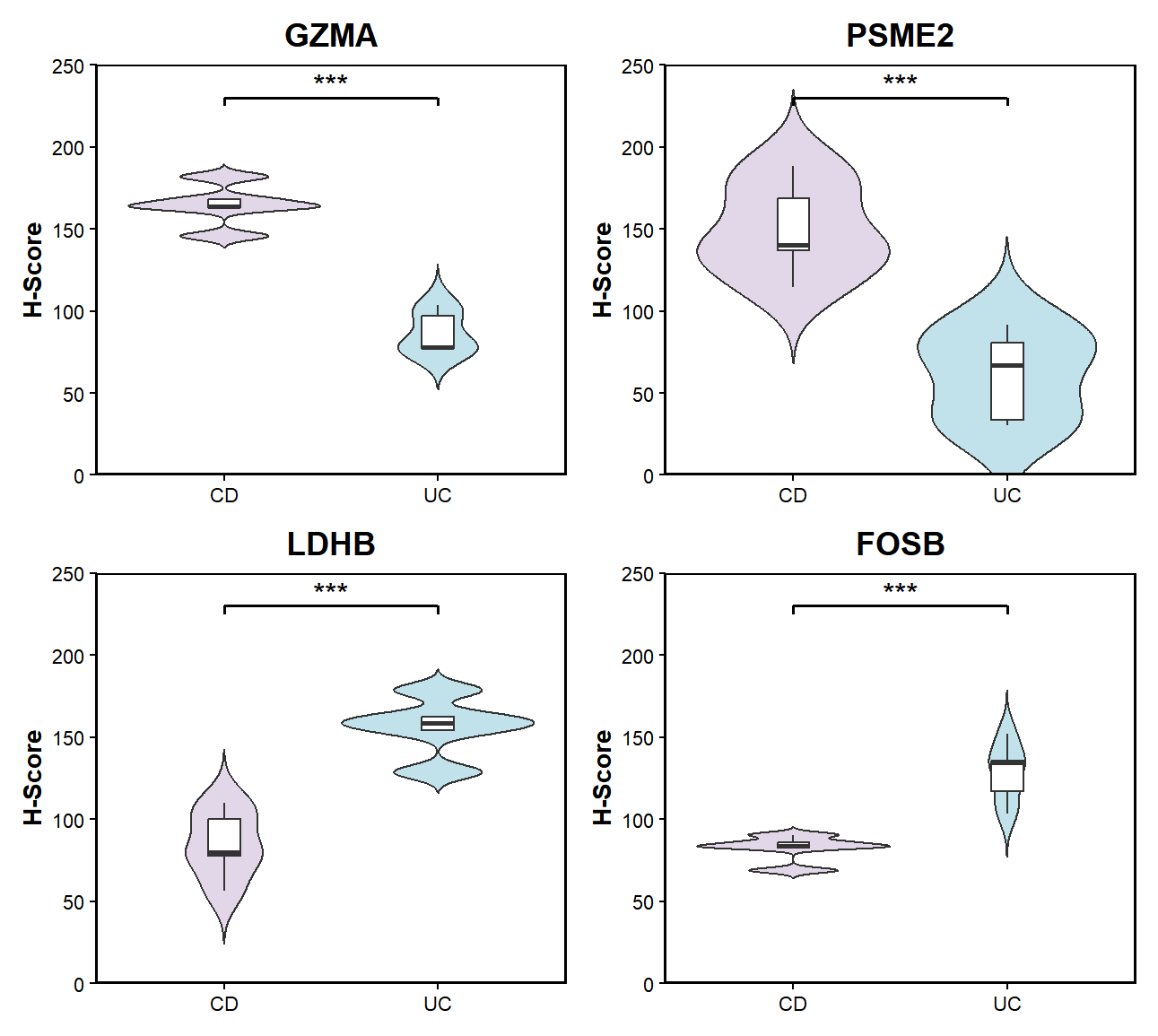

Supplement: Supplementary file 1 [file Supplementary_file_1.docx]
